# Photodynamic Therapy for Basal Cell Carcinoma: The Clinical Context for Future Research Priorities

**DOI:** 10.3390/molecules25225398

**Published:** 2020-11-18

**Authors:** Nicholas J. Collier, Lesley E. Rhodes

**Affiliations:** Photobiology Unit, Dermatology Centre, The University of Manchester & Salford Royal NHS Foundation Trust, Manchester Academic Health Science Centre, Manchester M6 8HD, UK; lesley.e.rhodes@manchester.ac.uk

**Keywords:** photodynamic therapy (PDT), basal cell carcinoma(s) (BCC), photosensitizer, protoporphyrin IX (PpIX), 5-aminolevulinic acid (ALA), methyl aminolevulinate (MAL)

## Abstract

Photodynamic therapy (PDT) is an established treatment option for low-risk basal cell carcinoma (BCC). BCC is the most common human cancer and also a convenient cancer in which to study PDT treatment. This review clarifies challenges to researchers evident from the clinical use of PDT in BCC treatment. It outlines the context of PDT and how PDT treatments for BCC have been developed hitherto. The sections examine the development of systemic and subsequently topical photosensitizers, light delivery regimens, and the use of PDT in different patient populations and subtypes of BCC. The outcomes of topical PDT are discussed in comparison with alternative treatments, and topical PDT applications in combination and adjuvant therapy are considered. The intention is to summarize the clinical relevance and expose areas of research need in the BCC context, ultimately to facilitate improvements in PDT treatment.

## 1. Introduction

Following its discovery at the beginning of the twentieth century, photodynamic therapy (PDT) has been developed as a cancer treatment, with useful applications in certain skin cancers and precursor lesions, including in low-risk basal cell carcinoma (BCC), where topical PDT is now an established treatment option licensed in more than 18 countries [1]. Although systemic PDT predated topical PDT, a pivotal development occurred in 1990 with the introduction of endogenous photosensitization by protoporphyrin IX (PpIX) by Kennedy and Pottier via topical application of the prodrug 5-aminolevulinic acid (ALA) [2,3]. This technique, as refined over the intervening years, remains the mainstay of cutaneous PDT today, with an established role in BCC treatment confirmed by randomized control trials (RCTs) [4]. The current review includes the evaluation of topical PDT applications in different patient populations and subtypes of BCC and comparison of the efficacy, cosmesis, and tolerability of topical PDT for BCC with alternative treatments, providing an overview of the current clinical position. Many ingenious ways of improving its effectiveness are being researched [5,6], and this review highlights the advances in knowledge of PDT for BCC treatment, including evidence from RCTs; it also draws attention to the gaps in the knowledge and indicates a range of relevant developments in research.

### Background, Aims, and Methods

This review of PDT for the treatment of BCC evolved from an earlier work by the authors on the guidelines for topical PDT [1] and a systematic review and meta-analysis of conventional and topical PDT for BCC and related works [4,7,8]. The current evaluation gives the opportunity to set the previous works in context, to include a wider range of publications, to shed light on the current limitations of PDT for the treatment of BCC, and to indicate promising areas of further research to address these.

This focused review is based on comprehensive searching of the literature, including those of the earlier works, from inception to the end of October 2020, using PubMed searches of papers written in English. Using the combination of search terms “basal cell carcinoma” and “photodynamic therapy” gave 672 results at the final search on the 1st November, 2020. The relevant papers were identified and analyzed, and their references were followed up and included as necessary. It was aimed at providing an outline of the current state of the knowledge based on the analysis of RCTs of topical PDT in BCC, together with a historical overview of its development. The intention was to furnish those engaged in scientific research in this area with the current clinical contexts, together with recent relevant research findings, to provide indications of further research needs and opportunities. Moreover, BCC and, in particular, superficial BCC (sBCC) can be advocated as a research model for the greater understanding of aspects of PDT treatment of cancer.

## 2. Research Development in BCC and PDT

### 2.1. BCC as a Model Cancer for Aspects of PDT Research

**Figure 1:**
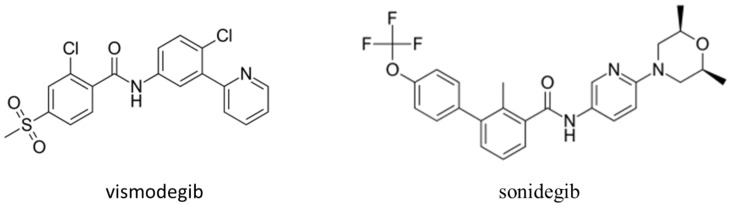
Sonic hedgehog inhibitors developed via research using basal cell carcinoma (BCC), and now, these molecules are used in the treatment not only of metastatic and locally advanced BCC but, also, in treatment and research in a wide range of cancers, including breast cancer and pancreatic cancer [21].

The most common human cancers are BCC, which comprise approximately 80% of the estimated 3.3 million keratinocyte carcinomas treated in the United States in 2012 [9,10,11]. A great deal of research in the molecular basis of BCC occurrence and development has been carried out, with the identification of important susceptibility genes [12,13,14,15,16], particularly with the unravelling of the dominant role of hedgehog signaling in the pathogenesis of BCC [16,17,18]. The initial report of the first clinical trial of topical ALA-PDT showed that BCC was used as the model for research, based both on its being the most common form of cancer in White Caucasians and its ready accessibility for both observation and treatment [2]. This has continued in the intervening years, and, for example, BCC has been advocated as a model in which to compare the expressed genes in normal and tumor cells with advantages of relative uniformity in histology and the absence of precursors [19]. The elucidation of the causes of genetic dermatoses such as Gorlin syndrome and xeroderma pigmentosum has highlighted the molecular mechanisms important in BCC, including the identification of associations between inherited common genetic variations in DNA repair genes and BCC development [20]. Developed in BCC studies, there has been uptake in research into, and the treatment of, cancers, generally, with development on approved hedgehog inhibitors, as illustrated in Figure 1.

There are many recognized unknowns both in BCC pathogenesis itself and in its treatment by PDT. Although the understanding of BCC pathogenesis has been advanced through revealing the major role of malfunctioning of the sonic hedgehog pathway, much remains to be clarified; for example, why metastasis is so rare in BCC and in understanding the molecular/genetic basis of the extensive range of BCC subtypes. In the PDT treatment of BCC, much remains to be uncovered in understanding the mechanisms of resistance to treatment and the characteristics of refractory/recurrent BCCs that limit the use of PDT while alternative treatment modalities, including surgery, are available. Future research priorities have been set out that cover BCC; these include improving the prediction of the response to treatment and finding how to tailor both the prevention and treatment of BCC to individual risk factors and needs [10].

There is an advantage in using sBCC as a research model for PDT treatment, as this allows the focus to be on those residual limitations of PDT treatment that remain when tissue penetration problems are minimized. Furthermore, although the incidence of all BCC is increasing, that of sBCC is increasing at a greater rate and affecting younger people, particularly females [9,22]. Care will be required to differentiate sBCC from other BCC subtypes with which it is often found, particularly in lesions of head and neck sites [22].

### 2.2. Mechanisms of Action of PDT

**Figure 2:**
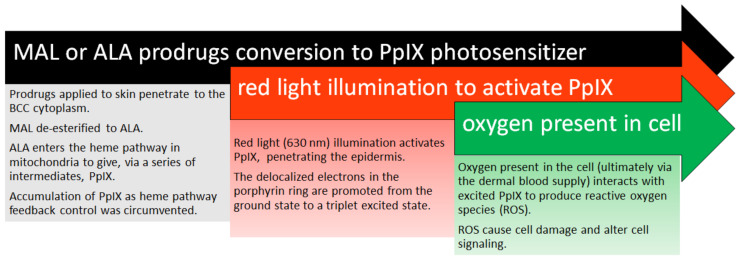
Topical photodynamic therapy mechanisms of action (MAL, methyl aminolevulinate; ALA, aminolevulinic acid; PpIX, protoporphyrin IX; BCC, basal cell carcinoma; and ROS, reactive oxygen species).

The three essential components of PDT are light and oxygen in the presence of a photosensitizer (Figure 2).

The development of PDT owes much to Dougherty et al., who developed exogenous photosensitizers based on hematoporphyrin and applied this methodology to a variety of human cancers, including BCC [23]. This work led to the first U.S. Food and Drug Administration approval of PDT. A brief history of Dougherty’s contribution was recently published [24]. Dougherty also produced an early account of the mechanisms of PDT in which, in addition to apoptosis and necrosis, immunomodulatory effects were cited [25]. He paid tribute to Kennedy’s highly innovative idea of photosensitizer ”delivery” via topical ALA [2,25]; ultimately there has been the widespread adoption of topical, rather than systemic, PDT for the treatment of BCC.

**Figure 3:**
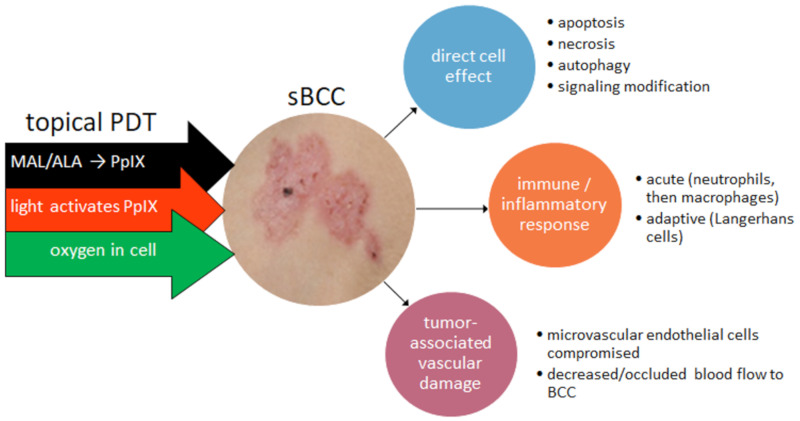
Effects of topical photodynamic therapy on superficial BCC (sBCC) (MAL, methyl aminolevulinate; ALA, aminolevulinic acid; PpIX, protoporphyrin IX; and BCC, basal cell carcinoma).

The principal photosensitizer currently widely used in BCC treatment is PpIX. This is endogenously produced following topical application to the BCC lesion of the prodrug; for example, 5-aminolevulinic acid (ALA) or its more lipophilic ester, methyl aminolevulinate (MAL). The prodrug undergoes relative selective uptake by the BCC cells compared with healthy tissue. Within the BCC cell, the ALA (to which MAL may be converted enzymatically) is metabolized in mitochondria via the heme biosynthetic pathway to PpIX, the active photosensitizer. The initiation of photosensitization follows, in which the PpIX molecule then absorbs light, causing a promotion of electrons from the ground state to produce a triplet excited state. In the presence of oxygen, the excited PpIX produces reactive oxygen species (ROS), which mediate cell damage and alter cell signaling and apoptosis or necrosis, as illustrated in Figure 3.

A study of the effect of ALA-PDT on the human immune system in BCC patients concluded that it was likely to lead to immune modifications contributing to treatment efficacy [26]. The ability of PDT treatments to enhance antitumor immunity in BCC patients in a clinical setting has been supported by a study involving both topical ALA-PDT and systemic porfimer sodium-PDT [27]. In this study, immune recognition of the BCC-associated tumor antigen, hedgehog-interacting protein (Hip1), was significantly greater than with surgically treated BCC patients. A greater effect was seen in superficial BCC (sBCC) than in nodular BCC (nBCC), with the immune reactivity being inversely related to a light dose and area of treatment [27].

A recent thermographic energy analysis of the topical MAL-PDT treatment of 17 sBCC and 23 nBCC lesions (all BCC lesions ≤10-mm diameters) was performed by monitoring with thermography and fluorescence imaging [28]. This found that most of the energy delivered was used in the photodynamic effect, and <1% was converted to local heating of the tissue, showing that the PDT thermal component was not sufficient to cause thermal damage [28].

A wide range of molecules have been investigated as photosensitizers with the intention of improving on the performance of currently approved photosensitizers for the treatment of tumors other than skin cancers—for example, rationally designed ruthenium(II) and iridium(III) complexes [29,30,31,32]. These developments are progressing through the preclinical stages, and one, TLD1433, has completed a phase 1b clinical trial (NCT03053635) involving six patients with non-muscle-invasive bladder cancer [33]. As yet, these developments have not, to our knowledge, been applied to BCC in preclinical trials, their potential use being directed to the eventual treatment of internal cancers, where their design may overcome problems such as the use of PDT in hypoxic environments [34,35]. It would be of interest to see studies of such compounds in the environment of BCC to compare them with the licensed topical prodrugs for PDT treatment.

In order to improve the penetration of PDT irradiation, near-infrared (NIR)-absorbing photosensitizers were synthesized and their photocytotoxicity recently demonstrated for the first time in highly pigmented B16F10 melanoma cells using NIR-Ru^II^ photosensitizers [36]. The three different ligands surrounding the central Ru^II^ were rationally chosen to achieve the objectives of moving the absorption into the NIR, facilitating the generation of singlet oxygen, and to control the solubility and stability of the complex [36]. All this series of compounds had absorptions at greater than 800 nm and showed strong effects at 733 nm in the highly pigmented melanoma cells tested [36]. Given the huge potential of modern chemical synthesis to produce such photosensitizers, it certainly seems possible that this may result in clinically useful outcomes. However, the application of rationally designed photosensitizers to the treatment of BCC via PDT is as yet unrealized; it would be of great interest to see them being tested in comparison with their currently clinically proven counterparts.

## 3. Drug Development in PDT for BCC

A turning point in the development of PDT occurred in 1960 at the Mayo Clinic with the observation of hematoporphyrin-induced fluorescence in neoplastic lesions, followed by the isolation of the porphyrin mixture “hematoporphyrin derivative” (HPD). This mixture was subsequently further purified to produce porfimer sodium, which has been widely used as a photosensitizer in systemic PDT [37].

The majority of BCC can be successfully treated by surgical excision, PDT, or other means. However, the less frequent highly challenging situations of locally advanced BCC and the exceedingly uncommon metastatic BCC can now be approached with systemic anticancer therapies, including hedgehog inhibitors and immunotherapy [38,39,40]. Additionally, those suffering from genetic conditions associated with the development of multiple BCC, such as Gorlin syndrome patients, may also benefit from systemic anticancer therapies [41,42,43], while topical and systemic PDT can be effective [41,42,43]. Resistance mechanisms of BCC to systemic therapies, including those involving microRNAs, is an emerging field of study [6]. The combination of systemic therapies with PDT provides an elegant treatment option for patients with multiple BCCs [44].

Topical PDT for BCC is an approved treatment in many countries. Though not yet approved in the USA, its use as an “off-label” treatment has generated interest, as indicated in a recent review [45]. For the topical PDT treatment of BCC, the prodrugs that are approved are ALA and MAL.

In many early topical PDT studies of the use of ALA, the prodrug solution was formulated in hospital pharmacies. These nonstandardized preparations may invalidate comparisons of the results from different studies [46]. The drug formulation of ALA is complicated by its ready dimerization to form pyrazine 2,5-dipropionic acid [47,48].

ALA-PDT should be considered in patients with nonaggressive, low-risk BCC, i.e., sBCC and nBCC not exceeding 2-mm tumor thickness, and where surgery is less suitable or contraindicated for lesion- or patient-related limitations (age and comorbidities, medications, logistic difficulties, position of lesions, such as poorly healing skin sites, and sBCC of large surface areas) [4,46]. A nanoemulsion gel formulation of ALA (BF-200 ALA) was developed and is described in Section 3.1 below.

Since 2006, MAL has been licensed in many countries for the treatment of low-risk BCC and is widely used, both in the clinic and in studies [1]. The licensed MAL-PDT protocol uses a cycle of two treatments separated by an interval of seven days, with a three-month follow-up [1,4,49].

Other prodrugs based on using ALA or its esters have been introduced; for example, hexyl aminolevulinate (HAL), which has been used for many years in urology for the detection of bladder cancer [50,51]. This longer chain ester prodrug was shown, in a recent RCT involving sBCC or thin nBCC, to have similar efficacy, tolerability, and cosmesis to both MAL and to BF-200 ALA [50]. Although many ALA esters were initially reported—particularly, n-alkyl esters (but, also, alkenyl esters)—it is not clear that the possibilities for further development have been exhausted; for example, esters formed from ALA and cholesterol or other steroids, or between ALA and penetrating moieties based on, for example, dimethyl sulfoxide, might offer benefits in tissue penetration [51,52]. In this spirit, a library of chemically diverse esters of ALA for testing in PDT was synthesized, using the multicomponent Passerini reaction, which offers an improved route to novel ALA esters and prodrugs [53]. Preliminary preclinical in vitro studies of two of these, 2-((4-fluorophenyl)amino)-2-oxoethyl-5-aminolevulinate and 2-(4-tolylamino)-2-oxoethyl-5-aminolevulinate, indicated improved properties for these esters compared with ALA [53]. Continuing research in these directions could potentially offer benefits in topical PDT, for instance, in reducing application times or enhancing penetration [54].

A brief summary of the remaining problems relating to the efficacy of the topical PDT treatment of BCC is provided in Table 1.

### 3.1. Nanoparticles

Materials in the nanorange 1–100 nm offer advantages in drug delivery and targeting, potentially resulting in increased efficacy and lower adverse effects of cancer therapies [55]. Liposomes are lipidic nanoparticles that were at the forefront of the nanomedicine drug delivery systems to achieve clinical use [56,57]. Known PDT photosensitizers have been incorporated into a nanoparticle (NP) platform, such as a micelle or liposome, to facilitate their delivery to tumors and improve phototoxicity [52]. Porphyrin combined with high-density lipoproteins (HDL) was developed into nanoparticles, which showed promise in preclinical studies in PDT for lung cancer therapy [53]. Here, the irradiation was by a 671-nm laser, and the porphyrin molecules were aggregated, at high density, with HDL in approximately 20-nm nanoscaffolds, which were biomimetic and allowed targeting of the tumor [53].

A nanoemulsion gel formulation of ALA (BF-200 ALA) was developed, and is used for sBCC or nBCC unsuited for surgical treatment due to possible treatment-related morbidity and poor cosmetic outcome [20]. This has been shown to have a similar efficacy to MAL in two recent RCTs, where the lesions treated were sBCC or thin nBCC [50,58]. In the larger multicenter RCT (281 patients), the BF-200 ALA-PDT was shown to be noninferior to conventional MAL-PDT, and both treatments were shown to have low recurrence rates of <10% when followed up at one year, together with high efficacy rates of >90% [50]. A further large trial of this nanoformulation, for use in sBCC treatment, is presently recruiting; some details are included in Section 7 below [59].

There are many examples where nanoparticles feature in approved treatments. One such is liposomal cytarabine (cytosine arabinoside) where the antimetabolite, which has low stability in plasma, is encapsulated within a lipidic bilayer. The encapsulation extends cytarabine’s half-life without the need for chemical modification of the cytarabine itself, where, like with ALA, esterification is an alternative [60,61]. This shows parallels, in the context of BCC, with ALA, where delivery of the zwitterion can be assisted by encapsulation as a nanoemulsion as an alternative to chemical modification via esterification. In the PDT context, all these are prodrugs for PpIX, but the ALA esters are prodrugs for the prodrug.

Nanoformulation with the potential for the topical treatment of BCC has moved forward with the development of ultra-deformable liposomes made of sodium cholate and soy phosphatidylcholine suited to penetration of the stratum corneum [62]. It has been shown that this nanovesicle can be loaded with the hedgehog inhibitor Vismodegib, which is approved for systemic use with certain very high-risk BCC, the reason for the restriction being the adverse effects arising from systemic administration; this nanoformulation may allow topical BCC treatment with reduced adverse effects [62,63].

## 4. Light Sources for PDT

Light-emitting diode (LED) lamps, halogen lamps, and lasers are used as light sources in PDT of BCC, as previously reviewed [64,65]. Red LED lamps ~632 nm are most often used, due to their convenience and narrower wavelength spectrum than halogen lamps; targeting of the photosensitizer at the required skin depth is therefore facilitated [66]. Lasers, which are more expensive and require higher maintenance, are also effective [67].

While the majority of BCC undergoing topical PDT are treated with LED lamps ~632 nm, and most patients tolerate the usually short-lived pain/discomfort accompanying the irradiation, there is interest in exploring other approaches with the objective of reducing the pain experienced. It has been demonstrated that daylight can be sufficient to allow MAL-PDT to function; the effects of weather conditions, season, and latitude have been assessed, showing the potential of daylight-mediated PDT (DL-PDT). It is now a frequent method of topical PDT for superficial precancerous skin lesions, i.e., thin-moderate actinic keratoses (AK) of the head and neck [68]. The use of DL-PDT for BCC has been investigated in a case series of 21 patients/32 lesions, where, after the application of MAL but, unlike conventional PDT, without an intervening period of occlusion, the BCC were exposed for 2.5 h to daylight. This resulted in a 74% clearance of BCC at one year [69]. The advantages include the marked reduction of pain during light exposure, although the exposure to daylight is considerably longer than the 7–9-min irradiation by LED at ~632nm [70].

A study comparing topical MAL-PDT using a low-irradiance ambulatory inorganic LED with conventional topical MAL-PDT in patients with a total of 30 sBCC found that low irradiance ambulatory PDT may be more convenient, as well as less painful, than conventional PDT, without a loss of efficacy and with an indication of improved patient preference [71]. A study of 223 sBCC, treated with either DL-PDT alone or with DL-MAL-PDT followed by imiquimod (IMQ), showed high mean response rates of 83.4% and 91.3%, respectively, after one year (*p* = 0.01) [72]; the full report on this study will be of interest. A cohort study recorded pain scores during the MAL-PDT treatment of 87 sBCC by different light deliveries [73]. Of these sBCC, 51 were treated with conventional MAL-PDT and 36 with a modified protocol using white light (LED, 75 J/cm^2^ at 55 mW/cm^2^); the time of exposure was varied by lesion size in the range of 83–93 min [73]. The white light treatment arm showed negligible pain, whereas the conventional PDT arm varied in the range of mild to severe pain; however, the potential of the development of artificial white light use in PDT is dependent on a demonstration of equivalency of lesion resolution in RCTs [73].

## 5. BCC Subtypes and PDT

BCC has no known precursor lesions, and a search of the literature confirmed this [19]. Here, the term “precursor lesion” is used to denote a clinically visible lesion capable of histological identification as a separate and preceding entity to the BCC itself, as opposed to a precursor cell or group of cells that are presumed to precede the development of a BCC in, for example, sun-damaged skin. The other main cutaneous keratinocyte carcinoma (KC), cutaneous squamous cell carcinoma (cSCC), in contrast, has, as precursor lesions, i.e., both AK, which are nonmalignant (although some authors regard AK as in-situ SCC [74]), and Bowen’s disease (BD), which is a malignant cancer in-situ precursor lesion of, but with relatively little (about 3–5%) progression to, cSCC [75]. In its growth pattern, sBCC has some resemblance to the cancer in-situ BD, but it is not itself a cancer in-situ, although both are epidermally limited, KC [76]. A retrospective cohort study found increased BCC numbers in cSCC patients, and this and other studies point to an overlap of risk factors and pathogenesis in different skin cancer types involving immunosuppression, UVR exposure, and genetic factors [77,78,79,80,81]. The ratio of incidence BCC:cSCC of the two KC varies from a predominance of BCC in the general population to the reverse in immunosuppressed organ transplant populations; this ratio is of importance in understanding these cancers, and a call has been recently made for uniformity in the calculation of this ratio [11].

It has been argued that the purpose of subtyping BCCs is to guide the treatment choice and not to distinguish biological differences, and for example, the sBCC designation defines a candidate of consideration for topical treatments [82]. It is important for researchers to realize that clinicians and histologists increasingly define subtypes with an overriding focus on treatment rather than biology.

The diagnosis of BCC is often made clinically and with histopathological confirmation. Whilst numerous BCC subtypes have been described, the more common subtypes with frequent agreement between dermatopathologists include nodular; superficial; infundibulocystic; fibroepithelial; and the aggressive growth variants such as infiltrative, micronodular, and morpheaform (sclerosing) [83,84,85,86].

The health “risk” associated with a BCC lesion is a function of both the BCC subtype and its location and size. Thus, although both sBCC and nBCC would normally be regarded as “low-risk” subtypes, larger thicker lesions in high-risk zones are treated as “high-risk”. Topical PDT can be an appropriate treatment for BCC lesions, where these are intrinsically low-risk BCC subtypes but are large or in high-risk sites, such as the facial “H”-zone, or in patients at high risk of surgical complications [87,88,89]. An RCT was carried out using MAL-PDT on such a high-risk nBCC. This compared conventional MAL-PDT with ablative fractional laser (AFL)-MAL-PDT to potentially improve MAL uptake [90]. However, the long-term efficacy was similar, and no clear advantage over conventional PDT was seen [90].

PDT treatment is limited by the depth of penetration of the radiation, which varies with both the depth and pigmentation of the BCC and with the wavelength of the radiation. Some of these newer photosensitizers have the ability to extend the range of PDT into the near-infrared (NIR), potentially offering the prospect of deeper penetration of the treatment in tissues and, also, to penetrate pigmentation, which limits the PDT efficacy both through the pigment absorbing the incident radiation and by the pigment quenching of the PDT-generated ROS.

### 5.1. Low-Risk BCC Subtypes

The sBCC and nBCC subtypes may exist in combination with other subtypes. Thus, incomplete clinical sampling or partial histologic evaluation may lead to missing the presence of a more aggressive subtype; recurrent BCC have a higher proportion of mixed subtypes [91,92,93].

The growth rate of sBCC has been estimated to be <1 mm^2^ per month, and it has been suggested that, due to this slow growth rate, modest delays in wait times are not likely to significantly adversely affect patient outcomes where the lesions occur in sites that are not cosmetically sensitive [94]. It would be of interest for research to be carried out to assess any increased risk of treatment failure arising from such delays. It is accepted that sBCC is the least concerning subtype [63], and it is found that sBCC show higher complete response rates to topical PDT than any other subtype of BCC; this is usually attributed to their relative thinness, allowing the easier penetration of both the light and prodrug [95,96]. However, although the incidence of BCC is increasing worldwide, the rate of increase appears to be greater for the sBCC subtype, with an increasing proportion being found at younger ages. This proportional increase in sBCC may, at least in part, be attributable to earlier detection.

As the penetration of both the prodrug and light decrease with the increasing depth of the BCC lesion, the recommendation to consider a treatment with topical PDT is typically limited, i.e., in the case of nBCC, to thin (<2 mm) nBCC [1].

### 5.2. High-Risk BCC Subtypes

Certain commonly occurring histological BCC subtypes show relatively aggressive tissue invasion and are categorized as high-risk; these include pigmented, morphoeic, micronodular, infiltrative, and basosquamous subtypes [97]. Topical PDT as an independent treatment for BCC is not recommended for high-risk BCC subtypes because of its lower clearance rate than the surgical treatment of BCC, and also, because the depth of penetration of both the prodrug and the activating light is limited [98]. This is a crucial issue in high-risk BCC subtypes, such as invasive BCC, where the depth of penetration is a critical requirement in effective treatment [98]. However, topical PDT may have a role as an adjuvant treatment to surgery in selected cases where a low-risk BCC subtype extends peripherally from a high-risk subtype. PDT treatment may be used prior to surgery in order to simplify the surgical task or, more commonly, following margin-controlled surgery, where an area of low-risk BCC remains and the risk of potential recurrence following PDT is outweighed by the morbidity from continued surgery [99,100].

## 6. Patient Population Subgroups

### 6.1. Solid Organ Transplant Recipients and Significantly Immunosuppressed Patients

Solid organ transplant recipients experience an increased risk of BCC of at least tenfold [101,102,103]. PDT is an option to treat these BCC, and, in view of the likelihood of repeated treatments, alternatives to surgery are of interest as a means of reducing adverse effects from the treatment and preserving the functionality and cosmesis [101,102,103]. PDT is an acceptable treatment for low-risk BCC in these patients, but, if used, ongoing surveillance is indicated to detect possible recurrences early [103]. In a retrospective study of 322 BCC in 23 solid organ transplant recipients and 80 nontransplant patients, recurrence was observed in 22.6% of transplant recipients versus 15.2% of nontransplant patients, although no significant difference was found between the two groups [104].

A retrospective chart review over an eight-year period of all patients who were treated in an Australian tertiary dermatology clinic with conventional topical MAL-PDT concluded that this treatment remains an appropriate second-line modality in organ transplant recipients [105].

### 6.2. Gorlin Syndrome Patients

Gorlin syndrome patients are subject to multiple BCC. Hence, if these are excised surgically, the extensive resulting scarring may be debilitating. PDT treatment reduces morbidity due to reduced scarring for these patients; moreover, wide fields containing multiple BCC are amenable to being treated noninvasively and simultaneously. A bilaterally controlled comparison pilot study of three patients with 141 BCC treated with ALA-PDT found >90% clearance of BCC at two months using irradiance with either blue or red lights [106]. The comparison between the blue and red light sources used for the ALA-PDT appeared equally safe and, apparently, equally effective [106]. Consensus recommendations produced from the personal experiences of seven experts, who treated a total of 83 patients with Gorlin syndrome using MAL-PDT, and, also, the results from a literature review citing nine relevant reports involving 59 patients, found MAL-PDT to be safe and generally effective [107]. Treatment was recommended for sBCC and nBCC, which were small, outside high-risk zones and after preparatory debulking. Anecdotally, MAL-PDT was preferred by patients in comparison with alternative treatments, including surgery, imiquimod, and 5-fluorouracil (5-FU) [107].

## 7. Outcomes of Topical PDT Treatment of BCC

A systematic review of topical PDT for BCC found this to be an effective treatment for low-risk BCC, and the outcomes are briefly summarized in Table 2 [4].

A jointly published pair of RCTs compared MAL-PDT with placebo cream-PDT [108]. This established the efficacy of topical PDT and showed better clearance at three months after the last treatment than placebo-PDT (risk ratio (RR) 2.75; 95% confidence interval (CI) 1.84–4.10), together with an improved cosmetic result (RR 3.00; 95% CI 1.80–5.01), although the MAL-PDT arms showed greater recorded manageable pain levels (RR 1.37; 95% CI 1.14–1.66) [4,108].

Topical PDT with ALA or MAL is one of several nonsurgical treatments available for low-risk nBCC and sBCC [118,119,120]. The conventional protocol for the treatment of BCC with MAL-PDT involves a double-treatment procedure with a one-week interval and a three-month clinical review where, if the BCC have only a partial response, they are usually retreated [49]. This procedure has been shown to give excellent cosmetic results, with higher clearance rates in sBCC than in nBCC [1,4,46,49]. Although this protocol is well-established, improvements in PDT outcomes via optimization of the methodology variables are being pursued, including improvements in the prodrug, prodrug delivery formulation, and light delivery [121].

**Figure 4:**
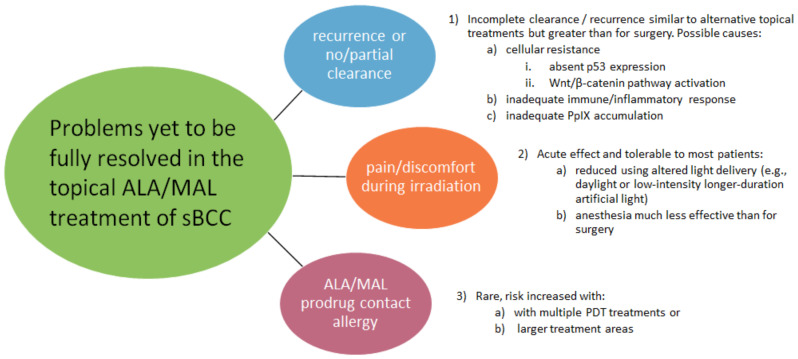
Schematic indicating the remaining problems in the topical photodynamic therapy (PDT) of superficial basal cell carcinoma (sBCC). (MAL, methyl aminolevulinate; ALA, aminolevulinic acid; and BCC, basal cell carcinoma).

The preferences of 124 patients for various aspects of BCC treatments were investigated. These patients ranked the first three outcomes in descending order of importance as the recurrence rate, cosmetic outcome, and cure rate [122]. Although sBCC is the most susceptible BCC subtype to topical PDT treatment and, being intrinsically thin, is relatively free from penetration problems of either light or the prodrug, remaining problems exist, and these are illustrated in Figure 4.

Pain/discomfort during irradiation is a prominent adverse effect of conventional topical PDT [4,8]. However, this does not usually prevent successful delivery of the treatment, although, in some cases, it may require the treatment to be paused and/or local anesthesia to be used [4,8]. Although pain levels have been reported to differ between the use of ALA and MAL, with less pain in MAL-PDT, a cohort study found no significant difference [123]. Optimizing the topical PDT treatment of BCC is fraught by the need to balance detriments; for example, the use of cold-air analgesia, while widely used and advocated [124], may compromise clearance [8,125]. Vasoconstriction hinders PDT, probably by reducing the necessary oxygen delivery, while surgical treatment benefits from vasoconstricting anesthesia. Problems relating to adverse effects, recurrence, and resistance encountered in the use of topical PDT for BCC treatment are briefly summarized in Table 3.

A negative outcome of topical PDT treatment is recurrence, which may sometimes be attributable to cellular resistance to the treatment. This is estimated to occur in up to 25% of cases, with several significant associations identified [126,127]. Those patients with p53-positive immunostaining were found to have an almost 69-times greater chance of a complete response to PDT than p53-negative patients [126]. In addition to absent p53 expression, this study also identified other indicators of poor PDT response, such as age >63 years, nBCC, β-catenin peripheral palisading of basal cell islands reinforcement, and the absence of peritumoral infiltrate [126]. Resistance mechanisms arising in BCC treatment with PDT have received little study hitherto; however, recent studies have identified associations with changes in p53 expression and in Wnt/β-catenin pathway activation, which have shed light on the resistance mechanisms. A better understanding of this may eventually lead to clinical improvements [127].

In cancer treatment, generally, there is a major problem with refractory/recurrent tumors, for example, in acute myeloid leukemia [61]. The impact of refractory/recurrent tumors, both on the patient and clinician, can increase the burden on healthcare systems. In the case of BCC treatment, refractory/recurrent tumors are a relatively less frequent problem, as good treatment alternatives are usually available. Rarely, this is not the case—for example, in locally advanced BCC, which commonly occur either through patient neglect or tumor recurrence following prior insufficient treatments. Trials in Gorlin syndrome patients showed that, although hedgehog inhibitor treatment reduced the tumor burden in these patients, when the treatment was interrupted, BCC recurred [63,128]. Although the resistance to BCC treatment is being intensively studied in respect to hedgehog inhibitor treatment, it is relatively neglected in PDT treatment for BCC. There would be a potential gain in studying the refractory/recurrent tumors that occur in the topical PDT treatment of BCC, perhaps particularly those arising in sBCC, where penetration problems of light and the prodrug are both at a minimum.

Even with sBCC, there may be some penetration problem with thicker lesions, and it may be wise to focus on instances of refractory/recurrent sBCC that are ≤0.4 mm in thickness, this having been shown to be a cut-off point in a correlation between the IMQ treatment of sBCC and treatment failure; in this study it was speculated that failures in topical treatments of “thick” sBCCs, including PDT treatment, may be due to deeper nonresponding tumor nests, presumably due to deficient penetration [82]. There are several effective topical treatments available for sBCC, but currently, none of these compete with surgery with respect to the rates of cures, although they have other advantages [129].

There is a multicenter (15 sites) randomized, double-blind, vehicle-controlled trial presently recruiting to evaluate the safety and efficacy of the nanoemulsion formulation of ALA (BF-200 ALA, Ameluz^®^) using irradiation from a particular LED lamp (BF-RhodoLED^®^) in the treatment of sBCC with PDT (ClinicalTrials.gov Identifier: NCT03573401) [59]. This type of trial would provide an ideal opportunity to further research of any refractory/recurrent tumors arising and to correlate these with patient characteristics in order to both make improvements in the treatment of such tumors and, also, to potentially screen patients prior to PDT, to advise them on the risks of treatment failure as applying to them as individuals. By such means, the cosmetic benefits of PDT could be more widely extended, and further insights into the technique gained of the potential use for the treatment of other, more threatening, cancers.

### 7.1. Comparisons of Topical PDT with Alternative Treatments for BCC

There is broad agreement from systematic reviews and international guidelines that surgical excision is a more effective treatment for BCC than topical PDT, but, where PDT use is appropriate, it often produces a better cosmetic outcome, in addition to being noninvasive [1,4,46,130,131,132,133,134,135]. The need to carefully compare alternative treatments was recently emphasized in a critical appraisal [136].

#### 7.1.1. Topical PDT Versus Cryosurgery

Systematic reviews have evaluated RCTs of cryosurgery versus topical PDT for the treatment of BCC [4,137]. Two PDT versus cryosurgery RCTs have been carried out, but the conventional PDT protocol was not used, as the usual second session of PDT one week later was omitted; both used ALA and not MAL [109,138]. In 2001, the RCT of ALA-PDT versus cryosurgery in nBCC and sBCC (PDT 47 lesions and cryosurgery 41 lesions) involved lesions distributed 54% on the trunk and 28% on the head and neck, with the remaining 18% on the limbs [138]. The cryosurgery involved two cycles, 25–30-s freeze, and 2–4-min thaw, and the PDT ALA 20% used a 635-nm laser, delivering 60 J/cm^2^ but with further sessions if there was an incomplete response [138]. This RCT concluded that, while the recurrence rates were comparable (25% ALA-PDT and 15% cryosurgery), and the pain/discomfort was similar in both treatments, the cosmetic outcome was superior and healing time shorter with PDT [138].

The second RCT compared MAL-PDT versus the cryosurgery of primary sBCC, with 105 patients in the cryosurgery arm and 114 in the MAL-PDT arm [109]. The cryosurgery consisted of ≤20-s freeze repeated two to three times, and the MAL-PDT was limited to a single session, with a further cycle only if clearance at three months was incomplete [109]. This showed no difference (*p* = 0.86) in the five‑year recurrence rates (20% with cryosurgery and 22% with MAL-PDT) but improved cosmesis with PDT. However, this was based on a per-protocol analysis, and a later intention-to-treat analysis showed that, at the five-year follow-up, the clearance was lower in a single-session MAL-PDT treatment than with cryosurgery (RR 0.72, 95% CI 0.55–0.95) [4]. In the MAL-PDT arm, 60% of patients achieved excellent cosmesis compared with only 16% in the cryosurgery arm (*p* = 0.00078) [109].

In conclusion, these clinical trials, comparing limited sessions of topical PDT with cryosurgery, indicated similar efficacy and adverse events with cryosurgery but superior cosmesis with topical PDT.

#### 7.1.2. Topical PDT Versus Surgical Excision

Surgical excision is often regarded as the standard treatment for high-risk BCC and is also used in the treatment of low-risk BCC, as it offers the lowest recurrence rates [97,139,140]. However, PDT and other noninvasive treatments are also appropriate for low-risk BCC of superficial depth, with a particular advantage for multiple and large surface area lesions or poorly healing skin sites, and a cosmetic outcome is demonstrably superior with PDT [91,92,93] A recent systematic review and network meta-analysis indicated that, while there was a clear advantage of lower recurrence rates of surgery over PDT, there was a moderate strength conclusion that PDT was associated with significantly better cosmetic outcomes than surgery [141,142].

Three RCTs compared excision surgery and topical PDT for nBCC [110,111,112,113,143], and another RCT compared them in sBCC [114]. Two of these RCTs used ALA [110,111,143] and two MAL [112,113,114] and various noncoherent and laser light sources. With regard to the differences in clearance rates between PDT and surgical excision, a meta-analysis of the nBCC results gave PDT a slightly lower three-monthly clearance (RR 0.94; 95% CI 0.89–0.99; *p* = 0.03), which increased slightly by 12 months (RR 0.90; 95% CI 0.84–0.97; *p* = 0.006). However, in the case of sBCC, PDT was shown to be not inferior in clearance at three months but was inferior at 12 months (RR 0.91; 95% CI 0.85–0.96; *p* = 0.001) [4]. The differences in recurrence rates between the treatment modalities showed that, for two of the nBCC studies—one using ALA and the other using MAL—PDT had more recurrences (pooled RR 13.19, 95% CI 2.58–67.37; *p* = 0.002), with a similar difference in recurrences in favor of excision in the sBCC RCT [4]. With regard to the cosmetic outcome as assessed at one year, the results showed a clear advantage of PDT in both subtypes (sBCC; RR 1.68, 95% CI 1.32–2.14; *p* <0.0001 and nBCC; RR 1.82, 95% CI 1.19–2.80; *p* = 0.006) [4,112,114]. The use of injected local anesthesia prior to surgical excision, compared to conventional PDT without prior local anesthesia, no doubt explains the difference in low-to-manageable pain, which was higher in MAL-PDT (RR 1.81, 95% CI 1.09–3.01; *p* = 0.02) [4,114].

#### 7.1.3. Topical PDT Versus Topical Treatments

Topical chemotherapy, i.e., 5-fluorouracil (5-FU), may be used for low-risk BCC, and cure rates of 70% are typical [4,144]. Topical application is typically twice-daily for a month, and local adverse effects are common, including redness, swelling, crusting, itching, and, rarely, wound infection. Treatment is impractical for lesions that cannot be easily reached by those patients without regular assistance [4,144].

The topical immunomodulator imiquimod (IMQ) is used for similar BCC lesions as 5-FU is and has a similar application method and frequency, but it shows somewhat better efficacy and can be used for nBCC, as well as sBCC, though with poorer efficacy in nBCC. IMQ has similar local adverse effects as 5-FU under clinical trial conditions, and systemic influenza-like symptoms can also occasionally occur [4,144].

In a triple-arm large RCT of topical treatments for sBCC, which did not permit the standard practice of repeat PDT treatment of partially responding lesions, MAL-PDT was compared with IMQ and 5-FU [115,116,117]. At five years, more cases treated with IMQ were tumor-free (80.5%; 95% CI = 74.0–85.6) in comparison with 5-FU (70.0%; 95% CI = 62.9–76.0) and MAL-PDT (62.7%; 95% CI = 55.3–69.2) [4,115,116,117].

## 8. Potential Improvements in Topical PDT

### 8.1. Physical Pretreatment of the Skin

Various techniques have been studied for physically pretreating the skin to facilitate the delivery of the prodrug to the target BCC cells in order to maximize the PpIX accumulation and thereby increase the efficacy of the PDT treatment. Surface scraping or debridement is usual with sBCC and often used for nBCC, but nBCC may also be debulked to reduce thickness. An intraindividual unblinded RCT compared different physical pretreatment methods by measuring the resulting PpIX fluorescence in normal skin [145]. This demonstrated that laser pretreatment using AFL gave the best results, but similar results were shown for curettage, microdermabrasion, and micro-needling [145]. Although this study involved normal skin and not BCC, a further four studies have been carried out on the AFL-assisted MAL-PDT treatment of BCC, three of these being RCTs [90,146,147,148]. These four studies involved 139 BCC lesions, but, although differences in the studies impeded direct comparisons, there was an indication that AFL-PDT may have advantages over conventional PDT, particularly for sBCC and thin nBCC (<2 mm), where high clearance rates were shown, but overall, the current evidence does not yet justify the use of AFL-PDT in BCC treatment in preference over conventional topical PDT [146,148,149].

A prospective study of improved prodrug delivery used a CO_2_ laser pretreatment of both sBCC (46 sBCC, using CO_2_-AFL) and nBCC (135 nBCC, using continuous super-pulsed CO_2_ laser). This involved MAL-PDT in two sessions of combined therapy one to two months apart and showed high clearance rates for both subtypes without visible recurrence, limited to 2.8% of the BCC in 10.7 months mean follow-up (range four to 18 months) [150].

Dermatography (medical tattooing) is used in surgical reconstruction and sometimes to improve the appearance of scars [151]. The use of dermatography to enhance MAL penetration was studied in animal models and in a Gorlin syndrome patient in whom six nBCC were treated with MAL (mixed with 0.015% EDTA) delivered by dermatography, resulting in clearance with no recurrence at 28 months [152].

### 8.2. Single-Day Fractionated Irradiation Protocol

A randomized multicenter pilot trial studied the treatment of sBCC with a second light fraction on the same day as the first fraction, with an interval of either 1 or 2 h between the fractions. One application of MAL was used in both study arms, with the first light fraction of 20 J/cm^2^ using the 630-nm Aktilite VR CL 128 lamp (Galderma SA, Lausanne, Switzerland) given at 3 h [153]. This was followed by the second fraction of 55 J/cm^2^ at 4 h post-prodrug in one study arm and 5 h in the other study arm [153]. The complete response rates at three and 12 months were 63.6% and 80% in the 3 and 4-h application groups and 70% and 100% in the 3 and 5-h application groups [153].

Four single-session (single visit) protocols (G1-G4) using MAL-PDT were compared with the conventional protocol (two sessions at a one-week interval; group Gc) in a study of 164 patients with a total of 194 nBCC and 63 sBCC, all of which were of ≤2-cm diameters [154]. For G1-G4, two light fractions were used throughout; the first fraction was at 3 h, and the second fraction varied with the four protocols [154]. In the four single-session protocols, both fractions were delivered on the same day, accompanied by MAL reapplication in G2-G4. The interval between MAL application and light irradiation, the drug-light interval (DLI), was varied between the protocol groups, as was the light energy delivered, as follows: G1, DLI 30 min, fluence100 J/cm^2^; G2, 60 min, 100 J/cm^2^; G3, 90 min, 100 J/cm^2^; G4, 90 min, 150 J/cm^2^; and Gc, 180 min, 150 J/cm^2^. Clinical and histological evaluation followed at 30 days. The highest clearance rate of 95.4% was with MAL reapplication prior to the second fraction and a dark interval of 90 min with light exposures of 150 J/cm² at 125 mW/cm²; full assessment of these protocol changes awaits further follow-up [154].

### 8.3. Optimizing PpIX Accumulation or Photobleaching

The damage to the BCC cells induced by PDT determines the efficacy of the treatment, and this damage is increased with increases in the extent of PpIX photobleaching [155]. Studies monitoring PpIX fluorescence during the MAL-PDT treatment of sBCC have indicated the importance of maximizing PpIX accumulation and photobleaching, with photobleaching in the first PDT session correlating strongly with clinical clearance at three months [156]. Lower PpIX accumulation and photobleaching was associated with increased age and lesions in the extremities; this reduction in photobleaching was associated with reduced lesion clearance [156]. The use of air cooling of the skin to reduce pain was found to lower PpIX photobleaching and was associated with reduced treatment efficacy [125,156]. In contrast, adjuvant agents are being examined for their ability to increase PpIX accumulation; this may include the advantage of increasing PpIX concentrations at deeper levels of the BCC, where the prodrug penetration is lower (see 8.4.3).

### 8.4. Combination and Adjuvant PDT

The alternative treatments available for BCC differ both in failure in the clinical response due to resistance processes and to the nature of these resistance processes and variables; these differences underlie the potential of combining alternative treatments with PDT to improve the effectiveness [157,158]. Further experimental studies have examined the means of improving the efficacy of PDT through its combination with therapies that do not themselves treat BCC but may improve the effectiveness of PDT, often mediated through the enhanced accumulation of PpIX in tumor cells. In addition to combining PDT with other modalities such as laser treatment and surgery, PDT has also been used to treat BCC in combination with the topical application of imiquimod [158].

#### 8.4.1. PDT and Imiquimod

Imiquimod is a topical immunomodulatory drug that is postulated to act through the activation of Toll-like receptor 7, which is licensed for the treatment for sBCC but, also, demonstrates lower but appreciable clearance rates in nBCC [159,160].

The combination of PDT and IMQ treatments for recurrent BCC was investigated in a double-blind, placebo-controlled RCT [161]. This RCT compared ALA-PDT and IMQ (22 patients) versus ALA-PDT and placebo-cream (10 patients) [161]. All 34 patients had single, recurrent, facial BCC (mean diameter 5 mm) and previously had their lesions unsuccessfully treated by either cryosurgery, laser therapy, or surgical excision [161]. The PDT treatment in both arms was repeated after 48 h [161]. The IMQ or placebo was topically applied 72 h later and then twice weekly before sleep for five weeks [161]. The lesions were clinically assessed, including a photodynamic diagnosis (PDD), from six weeks following the last treatment up to 14 months [161]. The PDT plus IMQ arm showed greater adverse effects, including edema and skin erosion, but cosmesis was very good in both arms [161]. The complete response was greater (75%; 18/24 patients) in the PDT plus IMQ arm than in the PDT plus placebo arm (60%; 6/10 patients), and the other patients showed a partial response [161]. This was a preliminary result, and the improvement in the IMQ arm did not achieve statistical significance (risk ratio 0.63, 95% CI 0.22–1.75; *p* = 0.37); however the results, even in the PDT plus placebo arm, were encouraging, given the recurrent nature of the BCC treated [4].

A recent retrospective study reviewed the sequential treatment of sBCC (*n* = 21) with two cycles of topical MAL-PDT followed by IMQ 5% cream applied Monday to Friday nightly for six weeks. The anatomical site comprised the trunk (*n* = 8), lower limbs (*n* = 5), and face (*n* = 8). The lesion sizes were >2cm in 7/21 lesions. All patients were followed up for at least 24 months (range 24–95), where recurrence was observed in two patients (10%). The cosmetic outcome was assessed to be excellent in 12/21 (57%), good in 8/21 (38%), and moderate in 1/21 (5%) [162]. It is of interest that, in a study of sBCC treatment failures with the topical therapies MAL-PDT, 5-FU, and IMQ, the proportion of early failures in more aggressive BCC subtypes appeared lower after IMQ (26.3%) compared with MAL-PDT (54.8%, *p* = 0.086) and 5-FU (66.7%, *p* = 0.011), possibly due to the more aggressive subtype component of the lesion responding more to IMQ than to the other two treatments [163].

A case study reported clinical clearance of a nasal nBCC in a 92-year-old man who was treated first with two courses of MAL-PDT, followed after by an interval of 10 days by topical IMQ treatment five times per week for three weeks [164]. The PDT treatment produced a partial response, with a reduction of more than 50% of the nBCC; the subsequent IMQ treatment produced clearance with no recurrence at 15 months [164].

#### 8.4.2. PDT and Surgery

Many BCC exhibit mixed histological subtypes with two or more growth patterns within the same lesion [91]. A suitable single treatment option may be chosen that will treat the highest-risk component, thereby also treating the lower-risk areas. An alternative approach that may be considered, especially in lesions that are predominately sBCC or thin nBCC, involves the treatment of low-risk areas and high-risk areas separately.

A single-center, single-blinded RCT involving 19 patients investigated the neoadjuvant use of PDT prior to Mohs micrographic surgery to investigate whether this would reduce the total surgical defect size or the number or Mohs stages required [165]. All BCC were located on the cheeks, nose, or forehead, measuring >1cm^2^. Infiltrative and morphoeic subtypes were excluded, with 79% of lesions being nBCC. Two treatment sessions of MAL-PDT were performed one week apart, and Mohs surgery was performed within 2–10 weeks PDT treatment. No significantly different results were reported, although there appeared a modest reduction in Mohs stages and defect size in the PDT-Mohs-treated group.

A patient with a large multifocal recurrent sBCC was treated with Mohs surgery, but, after six stages, about half of the peripheral margins showed sBCC to be still present, and the wound defect measured 12.5 cm × 9 cm [166]. As this was a sBCC and the wound large, the treatment was switched to MAL-PDT, given as an adjunctive treatment in two treatments one week apart [166]. There was no recurrence at six months, and unusually rapid wound re-epithelialization was observed [166].

A controlled study in healthy skin investigating wound healing following punch biopsies to the upper arm in 27 older men found that, while the healing time was lengthened with PDT treatment, the PDT-treated wounds appeared smaller, with improved collagen organization [167].

In IMQ therapy for sBCC, a retrospective study of 127 biopsy specimens found a correlation of treatment failure with thickness, with 58% recurrence for sBCC specimens >0.40-mm thick and no recurrence for those ≤0.40-mm thick at a mean of 34 months (range 3–91) [82].

#### 8.4.3. PpIX Enhancing Agents

**Figure 5:**
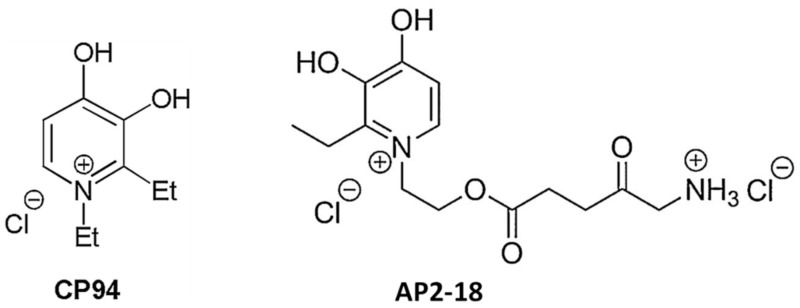
Molecular structures of the hydroxypyridinone iron chelator, CP94, 1,2-diethyl-3-hydroxypyridin-4-one, together with AP2–18; the aminolevulinic acid (ALA) prodrug developed from it, incorporating the iron chelator and the ALA prodrug in the same molecule [170,171,172].

In a controlled healthy human volunteer study, the impact of adding an iron chelator, desferrioxamine (DFO), to topical PDT using ALA was examined [168], the rationale being that the removal of iron reduces the conversion of PpIX to heme. The addition of DFO was found to significantly enhance both PpIX fluorescence pre-light exposure and phototoxicity (skin erythema) postexposure. In patients with two matched lesions, no improvement was seen in the clinical clearance of sBCC treated with DFO-ALA-PDT versus ALA-PDT, although the number of patients studied was small [119]. Early clinical investigation suggested a benefit with the iron chelator CP94 [169]; this has recently been developed into a novel iron-chelating ALA prodrug [170]; these molecules are illustrated in Figure 5.

Differentiation-promoting agents, including methotrexate, 5-FU, and vitamin D also modulate PpIX accumulation; a clinical trial (NCT03467789) is underway to examine whether oral vitamin D supplements can improve BCC treatment outcomes [5,173].

#### 8.4.4. PDT Enhanced by Epigallocatechin-3-Gallate (EGCG)

EGCG, a nontoxic catechin found in green tea (*Camellia sinensis*), has an established place in preclinical research on cancer prevention and treatment generally, with increasing clinical applications [174,175]. A MAL-PDT-resistant cSCC cell model was developed showing reduced PpIX and ROS generation, but these PDT-resistant cells were resensitized by EGCG, indicating its potential as an adjuvant to MAL-PDT [176]. In other cancers, EGCG has been shown to reduce cell growth better when combined with PDT compared with either treatment separately [177]. EGCG itself has been shown to inhibit cell cycle progression and promote apoptosis, in addition to enhancing the action of other chemotherapeutics such as 5-FU [178]. While normal cells are spared, EGCG causes apoptosis in cancer cells by a mechanism that in vitro studies suggest may be due to ROS production in those cells, together with the epigenetic triggering of apoptosis [179]. ECGC, in preclinical studies of cancer cells, was found to show the greatest antiproliferative effects of the various green tea components, inducing both blocking of the cell cycle and apoptosis, and had an even more potent antiproliferative effect than 5-FU [180].

If ECGC were to be tested in vivo as an adjuvant to topical PDT, consideration would need to be given to its careful formulation, as its lower activity found in in vivo compared with in vitro studies is attributable to its low stability [181]. Preclinical trials of the effect of EGCG on the PDT responsiveness on cancer cells (mouse mammary carcinoma cells and their transplanted tumors in mice treated with Photofrin-PDT) found that EGCG increased the apoptosis and cytotoxicity of the PDT treatment [182].

## 9. Conclusions

Over the last 40 years, topical PDT, using ALA or MAL, has been developed as an effective treatment for both sBCC and thin nBCC (<2-mm depth), with evidence based on a series of RCTs that have been systematically reviewed [4]. The sustained clearance rates are lower than with surgery, but the treatment is less invasive, and cosmesis is typically superior. Topical PDT is considered as an alternative to surgery for the treatment of sBCC and thin nBCC where surgery is less suitable for a particular patient or lesion, including poorly healing skin sites, multiple lesions, when cosmesis is important, and with patient preference.

For high-risk BCC, PDT is generally avoided, and there is no RCT evidence-based justification for its use where surgery is indicated [4]. However, there is some evidence, in case series and reports, of the successful use of PDT; regular and prolonged follow-up of the local site is required in these cases.

Improvements in the protocols of topical PDT for BCC continue to be researched. Current clinical evidence for enhancing the efficacy includes support for fractionated irradiation regimes and for combinations with AFL or topical therapies such as 5-FU and IMQ.

Further research to elucidate the causes of the refractory/recurrent behaviors of BCC following PDT treatment and, in particular, focusing on those occurring in carefully selected true sBCC may both extend the use of this technique in BCC and potentially assist in furthering PDT in other cancers.

## Figures and Tables

**Table 1:** Problems concerning the efficacy limitations of PDT for BCC treatment.

Problem	Consequences and Details	Sections
Clearance	Surgery often preferred, though inferior cosmesis. Not approved in some countries, including USA.	Section 3; Section 7.1.2. Section 3.
Subtypes	Restricted to nonaggressive, low-risk BCC.	Section 3.
Penetration	Even low-risk subtypes limited to thin (≤2mm) lesions. Penetration of prodrug and light decrease with lesion depth.	Section 3. Section 5.1.
PpIX accumulation	Need increased PpIX at deeper levels where penetration is lower. Adjuvant studies to increase PpIX accumulation. Iron chelators to reduce conversion of PpIX to heme. Differentiation promoters to improve PpIX accumulation.	Section 8.3. Section 8.3. Section 8.4.3. Section 8.4.3.

PDT, photodynamic therapy; BCC, basal cell carcinoma; and PpIX, protoporphyrin IX.

**Table 2:** Outcomes of topical PDT treatments of BCC from randomized control trials (RCT).

RCT	Prodrug	BCC Subtype	Clearance at 3 Months	Cosmesis
[108]	MAL	nBCC	73%	56% good-to-excellent
[109]	MAL	sBCC	90%	27% excellent
[110,111]	ALA	nBCC	93%	- (not assessed)
[112,113]	MAL	nBCC	91%	72% good-to-excellent
[114]	MAL	sBCC	87%	77% good-to-excellent
[115,116,117]	MAL	sBCC	82%	57% good-to-excellent
[90]	MAL	nBCC	88%	56% excellent

PDT, photodynamic therapy; BCC, basal cell carcinoma; MAL, methyl aminolevulinate; nBCC, nodular BCC; sBCC, superficial BCC; and ALA, 5-aminolaevulinic acid.

**Table 3:** Problems concerning adverse effects, recurrence, and resistance in PDT for BCC.

Problem	Consequences and Details	Sections
pain/discomfort during irradiation	Minimized in modified PDT regimens [1]. Local anesthesia less effective than for surgery. Cold air analgesia may reduce BCC clearance.	Section 4. Section 7.1.2. Section 8.3.
contact allergy to prodrug	Rare. Risk increased with multiple PDT/ larger treatment area [1].	[8]
recurrence	Rates significantly inferior to surgery. Rates similar to other nonsurgical treatments [4].	Section 3; Section 5.2; Section 6.1; Section 7; Section 7.1.1; Section 7.1.2; Section 8.1; Section 8.4.1; Section 8.4.2.
cellular resistance	This can cause recurrence. Associated with absent p53 expression. Associated with Wnt/β-catenin pathway activation.	Section 3; Section 7; Section 8.4.

PDT, photodynamic therapy and BCC, basal cell carcinoma.
